# Targeting fibrosis: mechanisms and clinical trials

**DOI:** 10.1038/s41392-022-01070-3

**Published:** 2022-06-30

**Authors:** Manyu Zhao, Liqun Wang, Mengzhu Wang, Shijie Zhou, Ying Lu, Huijie Cui, Alexandra C. Racanelli, Ling Zhang, Tinghong Ye, Bisen Ding, Ben Zhang, Jinliang Yang, Yuqin Yao

**Affiliations:** 1 West China School of Public Health and West China Fourth Hospital, Sichuan University, Chengdu, 610041 China; 2 State Key Laboratory of Biotherapy and Cancer Center, West China Hospital, Sichuan University, Chengdu, 610041 China; 3 Division of Pulmonary and Critical Care Medicine, Joan and Sanford I. Weill Department of Medicine, Weill Cornell Medicine, New York, NY 10021 USA; 4 NewYork-Presbyterian Hospital, Weill Cornell Medicine, New York, NY 10021 USA; 5 College of Polymer Science and Engineering, Sichuan university, Chengdu, 610000 China; 6 Key Laboratory of Birth Defects of MOE, State Key Laboratory of Biotherapy, West China Second University Hospital, Sichuan University, Chengdu, 610041 China

**Keywords:** Drug development, Target identification

## Abstract

Fibrosis is characterized by the excessive extracellular matrix deposition due to dysregulated wound and connective tissue repair response. Multiple organs can develop fibrosis, including the liver, kidney, heart, and lung. Fibrosis such as liver cirrhosis, idiopathic pulmonary fibrosis, and cystic fibrosis caused substantial disease burden. Persistent abnormal activation of myofibroblasts mediated by various signals, such as transforming growth factor, platelet-derived growth factor, and fibroblast growh factor, has been recongized as a major event in the occurrence and progression of fibrosis. Although the mechanisms driving organ-specific fibrosis have not been fully elucidated, drugs targeting these identified aberrant signals have achieved potent anti-fibrotic efficacy in clinical trials. In this review, we briefly introduce the aetiology and epidemiology of several fibrosis diseases, including liver fibrosis, kidney fibrosis, cardiac fibrosis, and pulmonary fibrosis. Then, we summarise the abnormal cells (epithelial cells, endothelial cells, immune cells, and fibroblasts) and their interactions in fibrosis. In addition, we also focus on the aberrant signaling pathways and therapeutic targets that regulate myofibroblast activation, extracellular matrix cross-linking, metabolism, and inflammation in fibrosis. Finally, we discuss the anti-fibrotic drugs based on their targets and clinical trials. This review provides reference for further research on fibrosis mechanism, drug development, and clinical trials.

## Introduction

Fibrosis is an important cause of global morbidity and mortality. Common diseases associated with fibrosis include hepatitis virus, nonalcoholic fatty liver disease (NAFLD), chronic kidney diseases, idiopathic pulmonary fibrosis (IPF), pneumonconiosis, and cystic fibrosis. The annual combined incidence of major fibrosis-related diseases is approximately 4968 per 100,000 person-years, causing huge disease burden^1^. Fibrosis-related diseases accounted for a large proportion of global disability-adjusted life-years (DALYs) in 2019^2^. Therefore, fibrosis is increasingly recognized as a major health challenge.

**Fig. 1:**
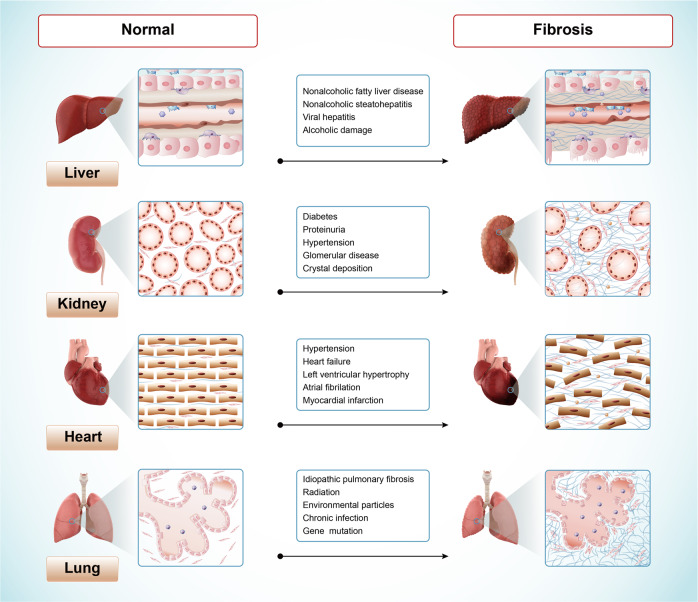
The aetiology of fibrosis in different tissues or organs

The normal wound healing process and the pathogenesis of fibrotic diseases share many mechanisms in common^3^. Various factors, such as infectious agents, alcohol, environmental particles, and gene mutation, can cause damage to normal tissue structures, triggering a wound-healing response^4^. The tissue repair response often starts with inflammation. Activated inflammation contributes to the upregulation of inflammatory mediators and promotes the migration of neutrophils, eosinophils, and macrophages to the injured site to clear debris and necrotic areas. Fibroblasts and other mesenchymel cells are then thansformed to myofibroblasts via the upregulation of fibrotic cytokines such as fibroblast growth factors (FGFs) and platelet-derived growth factor (PDGFs), which secrete extracellular matrix (ECM) components^5^. In normal wound healing response, activated myofibroblasts would be cleared from wound site via apoptosis after injury repair^6,7^. However, in fibrotic process, myofibroblasts fail to undergo apoptosis and are continuously activated, eventually leading to excessive ECM deposition^8^. The progressive accumulation of ECM leads to increased stiffness of injured tissue and hinders oxygen diffusion^9^, and further promotes cell damage. In addition, dysfunction of other parenchymal cells and dysregulated cell-cell interaction caused by injury are also the important causes of fibrosis, such as vascular proliferation induced by abnormal function of vascular endothelial cells^10^. The fibrotic process can occur in many organs, with fibrosis of liver, lung, kidney, and heart accounting for a large proportion of all fibrotic diseases^1,11^. The different characteristics of tissue structure and microenvironment between these organs lead to differences in the fibrotic process (Fig. 1). Despite increasing in-depth research on fibrosis, the mechanisms have not been fully explained, thus hindering the advancement of targeted drug research for fibrosis.

In this review, we briefly introduce the aetiology and epidemiology of several fibrosis-related diseases, including liver fibrosis, renal fibrosis, heart fibrosis, lung fibrosis, cystic fibrosis, and myelofibrosis. We then focus on the abnormal cells, aberrant signaling pathways, and anti-fibrotic drugs in fibrosis, providing reference for the mechanism and drugs research of fibrosis.

## Aetiology and epidemiology

### Liver fibrosis

Liver fibrosis, as a pathophysiological result of chronic liver injury, is the leading cause of mortality from chronic liver diseases (CLDs) worldwide. CLDs mainly include chronic infection with hepatitis virus, NAFLD, alcoholic liver diseases, and autoimmune liver diseases^12,13^. CLDs could progress to advanced liver fibrosis and eventually to cirrhosis^14^, which is the 11th cause of global death^15^. Hepatitis B virus (HBV), hepatitis C virus (HCV), and alcohol are the most common causes of DALYs from cirrhosis^16^. Alcoholic-related liver cirrhosis and other chronic liver diseases resulted in 332,300 all-age deaths and 9,785,400 years of life lost (YLLs) in 2017^17^. The prevalence of NAFLD is around 25% worldwide^18^, and its advance can progress to nonalcoholic steatohepatitis (NASH)^19^. NASH-related cirrhosis caused 118,000 all-age deaths and 3,285,500 YLLs in 2017^17^.

### Renal fibrosis

Renal fibrosis is caused by the damage to normal renal tubules, which eventually leads to glomerulosclerosis, tubulointerstitial fibrosis, and angiosclerosis^20^. Renal fibrosis is not a direct clinical diagnosis but a progressive and irreversible pathological feature of all chronic kidney diseases (CKDs)^21,22^. In 2017, CKDs caused 35.8 million DALYs, nearly a third of which were diabetic nephropathy^23^.

### Cardiac fibrosis

Cardiac fibrosis manifests as either reactive interstitial fibrosis and replacement fibrosis^24^. Reactive interstitial fibrosis refers to the expansion of interstitial and perivascular spaces without significant loss of cardiomyocytes and fundamental changes in muscle bundle structure^25^. Replacement fibrosis replaces dead cardiomyocytes with extracellular matrix tissue and fibroblasts, disrupting the continuous pattern of muscle bundles but maintaining tissue integrity^26^. Replacement fibrosis mainly occurs in response to ischaemia, ischaemia/reperfusion, inflammation, and toxic injury. Cardiac fibrosis is a common pathophysiological manifestation of most cardiovascular diseases, which are the leading cause of death, morbidity, and disability in most contries^27,28^.

### Lung fibrosis

The causes of chronic respiratory diseases are varied, including allergens, chemicals, radiation, microbial agents, and environmental particles^29^. Lung fibrosis is the main clinical outcome of most chronic respiratory diseases, such as pneumoconiosis and IPF^30^. IPF is the most common interstitial lung fibrosis with unknown aetiology^31,32^. The prevalence of IPF varies widely across regions, ranging from 0.33 to 2.51 in Europe, 0.57 to 4.51 in Asia-Pacific countries, and 2.40 to 2.98 in North America^33^. IPF mainly occurs in elderly individuals, with high mortality and morbidity^34,35^. Pneumoconiosis is a major occupational diseases caused by the prolonged inhalation of inorganic particles at work^36–38^. In 2017, all-age deaths of pneumoconiosis was 21,600 and 426,900 YLLs^17^.

### Cystic fibrosis

Cystic fibrosis is an autosomal recessive disorder mainly caused by mutations in the cystic fibrosis transmembrane conductance regulatory protein (CFTR) gene^39^. Compared with the high incidence rate of cystic fibrosis in Caucasians, cystic fibrosis was much less common in Asia, and the incidence rate varied from 1:10,000 to 1:40,750 among countries^40–42^.

### Myelofibrosis

Myelofibrosis, a myeloproliferative tumour with collagen deposition in bone marrow and splenomegaly, has low morbidity and shortened life expectancy^43–45^. Aberrant activity of the Janus kinase (JAK) /signal transducer and activator of transcription (STAT) pathway contributes to myelofibrosis^43,46^.

## Abnormal cells involved in fibrosis

Fibrosis is the result of the interaction between a variety of cells. Cell maps of fibrosis such as IPF, liver fibrosis, renal fibrosis, and systemic sclerosis have been well studied via single-cell sequencing^47–50^. These studies confirmed the key role of epithelial cells, endotheliocytes, immunocytes, and fibroblasts in fibrosis, and identified some new cell types involved in the pathological progress. This section will review the major cell types in fibrotic diseases.

### Epithelial cells

Epithelial cells, including basal cells, secretory cells, club cells, ciliated cells, and goblet cells, are essential cells to maintain tissue homeostasis in many organs^51^. In fibrotic process, chronic injury resulted in the apoptosis of epithelial cells, thus destroying the epithelial structure, promoting dysfunctional repair and pathogenic activation of fibroblasts^52^. Moreover, the epithelial-mesenchymal transition (EMT) is recognized as an important source of myofibroblasts. EMT under pathological conditions can lead to the reduction of normal epithelial cells, destroy the normal structure of the tissue, and promote the production of collagen fibers^53^.

Studies have showed that epithelial cells, such as alveolar epithelial cells, goblet cells, ciliated cells, and club cells, are crucial for the development of lung fibrosis^54,55^. Alveolar epithelial cells, including alveolar type 1 epithelial (AT1) and AT2 cells, are one of the main epithelial cells in lung tissue and maintain the integrity of the alveolar wall. When the injury leads to the death of AT1 cells, AT2 cells proliferate and differentiate into AT1 cells, so that the normal structural of the alveoli is maintained^56^. A new epithelial cell subset Axin2^+^ AT2 cells with both progenitor and epithelial properties was found in lung and regulate alveolar regeneration^57,58^. AT2-transdifferentiated plastic keratin 5 basal cells were co-located with pathological transforming growth factor (TGF) -β1^hi^ collagen triple helix repeat containing 1 (CTHRC1)^hi^ fibroblasts and have a synergistic effect in the progress of fibrosis^59^.

A new group of epithelial cells with high expression of CFTR, named ionocytes, was found in airway epithelium^60^. One of the most important functions of CFTR is to regulate chloride channels^61^. Therefore, the mutations of CFTR gene of epithelial cells results in chloride channel defects in airway epithelium, initiating the occurrence of cystic fibrosis^62^. Moreover, the lack of CFTR in airway increased Na^+^ channel activity and Na^+^ hyperabsorption, suggesting that CFTR might be involved in Na^+^ transport^61^. The functional change of epithelial cells in the pancreas and liver is also affected by CFTR mutation^63^. In the normal liver, CFTR cooperates with the chloride channel at the top of cholangiocytes to provide a driving force for bile hydration^64^. Impaired CFTR function lead to mucosal hyperplasia and obstruction of the bile duct. Subsequent bile salt accumulation contributed to hepatocyte damage, inflammation, and fibrosis in the portal vein^64,65^.

### Endothelial cell

Endothelial cells are main components of blood vessels. Damage to endothelial cells cause abnormal substances exchange between blood and tissues, resulting in metabolic disorders. Furthermore, in fibrotic tissues, abnormal angiogenesis may be induced due to the massive proliferation of fibroblasts requiring more blood nutrients. Studies showed that endothelial cells of different fibrotic tissues may also have specific functions. Two new endothelial cell subtypes, plasmalemma vesicle associated protein (PLVAP)^+^ endothelial cells and atypical chemokine receptor 1 (ACKR1)^+^ endothelial cells, were found in liver tissues of patients with liver cirrhosis and could promote the migration of leukocyte^48^. In lung tissues, five endothelial cell groups were identified by single-cell sequencing, including capillary endothelial cells A and B, venous endothelial cells, and arterial endothelial cells. The fifth kind of endothelial cells recognized by high expression of Collagen 15a1 (COL15A1) gene, located in the bronchioles and fibrous foci, was involved in the production of extracellular matrix^47^.

### Immune cells

Abnormality of immune system might be an early event of fibrosis^66^. Immunocytes, such as T lymphocytes, macrophages, dendritic cells, granulocytes, and mast cells, are involved in the fibrosis progress^49,67–70^. These activated immune cells highly express factors that regulate inflammation and fibrosis, promoting the activation of fibroblasts. T lymphocytes, including CD4^+^T cells, CD8^+^T cells, and CD8^+^effector cells, were increased in IPF patients^71^. The interferon-γ signal transduction in T lymphocytes in IPF was significantly changed^71^, while interleukin (IL) -6 signal in T lymphocytes was mainly up-regulated in patients with systemic sclerosis^67^. In liver tissues, the expression of cytotoxic T cells increased and the inactivation of CD4^+^ T cells could induce fibrosis^72^.

Macrophages are key cells that mediate inflammation and fibrosis in fibrotic diseases. Seven macrophage subsets were identified in the tissues of patients with liver cirrhosis, including Kupffer cells (resident macrophages in liver) and CD9^+^ triggering receptor expressed on myeloid cells 2 (TREM2)^+^ macrophages. Pseudo-time sequence analysis showed that TREM2^+^CD9^+^ macrophages were derived from monocytes and increased collagen expression in hepatic stellate cells (HSCs)^48^. In the lung fibrosis, 18 types of immune cells were found, and the phenotypes of tissue resident macrophages, fibrogenic macrophages and inflammatory macrophages were identified^47,54^. Resident macrophages in lung are mainly alveolar macrophages (AMs). AMs adheres closely to alveolar epithelium and are exposed to the outside environment^73^. Inhalable particles and other factors directly led to the death of AMs^74^. Activated AMs secreted inflammatory mediators to activate the inflammatory response, and elevated pro-fibrotic factors expression to promote lung fibrosis^75,76^. The sialic acid binding Ig-like lectin F (SiglecF)^+^ C-X3-C motif chemokine receptor 1 (CX3CR1)^+^ macrophages were also identified in pulmonary fibrosis mouse model, which were adjacent to fibroblasts and promoted fibrosis by releasing PDGFs to drive the proliferation and activation of fibroblasts^77^.

### Fibroblasts

Differentiation of fibroblasts to myofibroblasts with secretory, contractile, and extracellular matrix-producing properties is a key cellular event in many fibrotic conditions. Single-cell sequencing has demonstrated that myofibroblasts have different gene expression profiles with dynamic changes in fibrosis of different organs^78,79^. In lung tissue, the differentiation pathways of fibroblasts differ between normal and fibrotic pathological states. Mesenchymal progenitor cells differentiate into lipofibroblasts and COL14A1^+^ matrix fibroblasts, and the latter then differentiate into myofibroblasts and COL13A1^+^ matrix fibroblasts. In lung fibrosis, mesenchymal progenitors differentiate into lipofibroblasts, PDGFRβ^hi^ subtypes, COL14A1^+^ matrix fibroblasts, myofibroblasts, and COL13A1^+^ matrix fibroblasts^80^. The dominant cell type of fibroblasts in liver are HSCs, which are characterized by their star-like morphology. The differentiation of HSCs may undergo four processes: loss of quiescent properties, promoting inflammation, migration, and ECM production^50^.

Increasing number and activation of myofibroblasts induced by immune cells, EMT, and endothelial-mesenchymal transition (EndMT) are considered major contributors to the process of fibrogenesis^81,82^. Inhibiting the proliferation and activation of myofibroblasts has been a critical issue for the treatment of most fibrosis. However, in the fibrotic process, myofibroblast cells could obtain apoptosis resistance during differentiation^83^, which hinders the implementation of programmed death mechanisms^8^. Therefore, the therapeutic method for reducing the number of myofibroblasts has limited efficacy. Moreover, the hyper-activation of myofibroblasts is usually a compensatory result of the death of parenchymal cells such as epithelial cells, cardiomyocytes, and endotheliocytes. Therefore, it might be a more effective treatment method to decrease the death or modulate the activity of parenchymal cells and other related cells, so as to indirectly inhibit myofibroblast activation.

**Fig. 2:**
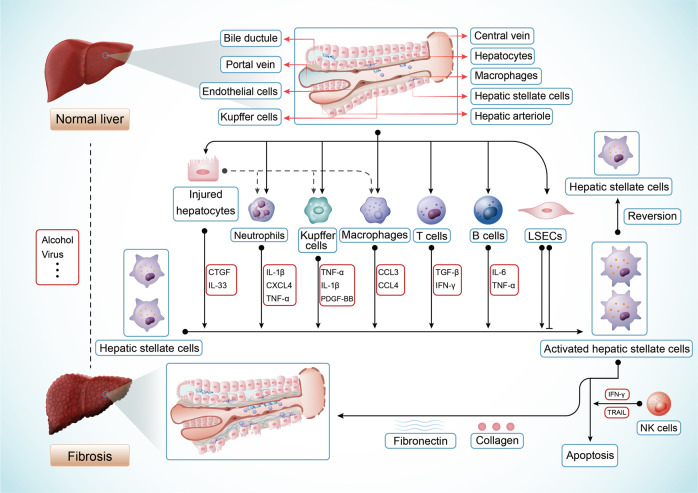
The activation of HSCs regulated by other cells in liver fibrosis. Extracellular components from injured hepatocytes, Kupffer cells, macrophages, NK cells, T and B lymphocytes modulate HSCs activation via various cytokines. LSECs inhibit or promote the activation of HSCs in different conditions. NK cells kill activated HSCs in IFNγ and TRAIL-dependent ways. TRAIL, TNF-related apoptosis-inducing ligand

In liver fibrosis, the interaction mechanism of HSCs with other cells is complex. Maintenance of liver sinusoidal endothelial cells (LSECs) differentiation leads to HSCs quiescence and fibrosis regression in normal liver^84,85^. However, in fibrotic process, apoptotic hepatocytes increase the inflammatory response and activate macrophages^86^. Extracellular events from Kupffer cells (liver-resident macrophages), hepatocytes, B lymphocytes, and T lymphocytes further modulate the activation of HSCs^87,88^. NK cells could kill activated HSCs via regulating retinoic acid-induced 1/natural killer group 2D (NKG2D) -dependent and TNF-related apoptosis-inducing ligands^89,90^. Chronic liver injury leads to continuous HSCs activation, which promotes ECM accumulation and tissue structure remodeling, and then results in progressive liver fibrosis^91^ (Fig. 2).

**Fig. 3:**
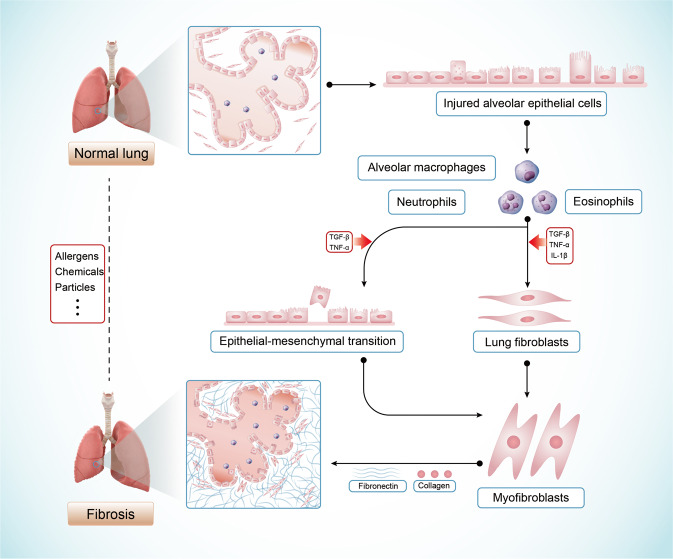
The interactions among cells involved in lung fibrosis. Injured alveolar epithelial cells activate macrophages, neutrophils, and eosinophils, resulting in the secretion of cytokines, such as TGF-β, IL-1β, and TNF-α. These cytokines mediate the differentiation of fibroblasts into myofibroblasts and the epithelial-mesenchymal transition, which result in the ECM deposition at the injury site

In the lung, acute injury of alveolar epithelial cells can cause the reduction of epithelial cells, the destruction of alveolar structure, and the release of pro-inflammatory mediators, thus activating immune cells. These activated inflammatory cells and injured epithelial cells increase the upregulation of cytokines, including TNF-α, IL-1β, IL-6, and TGF-β^92–94^. After the initial inflammatory events, pulmonary fibroblasts are activated into myofibroblasts by upregulating fibrotic cytokines such as PDGFs, FGFs, and vascular endothelial growth factor (VEGFs)^95–98^. The transition of epithelial cells by the EMT process could also increase the population of myofibroblasts. Chronic activated myofibroblasts produce ECM components (collagens, fibronectin, proteoglycan), leading to lung fibrosis^12,22,99^ (Fig. 3).

## Important signaling pathways in fibrosis

An overwhelming number of mediators have been implicated in fibrosis, regulating myofibroblast activation, metabolism, inflammation, and ECM cross-linking. This part mainly focus on the important signaling pathways involved in fibrotic diseases based on the research intensity and drug efficacy of drug targets in clinical trials.

### Growth factors and associated signaling pathways

**Fig. 4:**
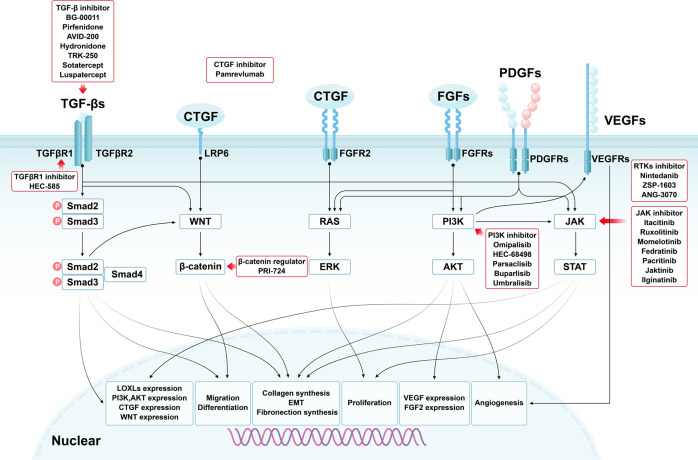
Interactions between growth factors-associated signaling pathways and a summary of related target drugs. PDGFs binding to PDGFRs activates the JAK/STAT, PI3K/AKT, and RAS/ERK signals. FGFs binding to FGFRs activates PI3K/AKT and RAS/ERK signals. CTGF binding to FGFR2 (promoting FGF2 and FGF4 binding to FGFR2) activates RAS/ERK signaling, and CTGF binding to LRP6 activates WNT/β-catenin signaling. Drugs targeting these signaling pathways are listed. EMT: epithelial-mesenchymal transition

The growth factors and associated signaling pathways have been reported to promote fibrosis by regulating fibroblasts activation, epithelial cells apoptosis, EMT, and EndMT. Growth factors mainly include TGF-βs, PDGFs, FGFs, and connective tissue growth factor (CTGF). Pathways, such as phosphatidylinositol 3-kinase (PI3K) / protein kinase B (AKT), JAK/STAT, and WNT/β-catenin, are the common downstream signals of these growth factors involved in fibrosis. The interactions between these signaling pathways in fibrosis are depicted in Fig. 4.

#### TGF-β signaling pathway

##### TGF-β activation

TGF-βs are the key cytokines in most fibrosis. There are three isoforms of TGF-βs, namely, TGF-β1, TGF-β2, and TGF-β3. The pro-TGF-β monomer synthesized in ribosome, folds in the lumen of the endoplasmic reticulum (ER) and dimerizes via a disulfide linkage. Then, the latency-associated peptide (LAP) binds to mature TGF-β and attaches to latent TGF-β binding protein (LTBP)^100^. This TGF-β/LAP/LTBP complex binds to the ECM in the extracellular space and inactivates TGF-β^101^. The complex can be cleaved by various proteases to release active TGF-β^102^. Activated TGF-βs bind to TGFβR2 and TGFβR1^100^. Upon ligand binding, phosphorylated TGFβR2 then phosphorylates and activates TGFβR1. Factors, such as epidermal growth factor (EGF), IL-1, and TNF-α promote TGF‑β expression in different types of cells^103,104^. Moreover, the precursors of TGF-β contain an arginine-glycine-aspartate (RGD) motif, which can be recognized by integrin αv/β6^105,106^, suggesting that the activation of TGF-β gene could be regulated by integrin αv/β6. Partial inhibition of TGF-β with an integrin αv/β6 antibody effectively prevented pulmonary fibrosis in mice without aggravating inflammation^107,108^.

##### Canonical and non-canonical signaling

**Fig. 5:**
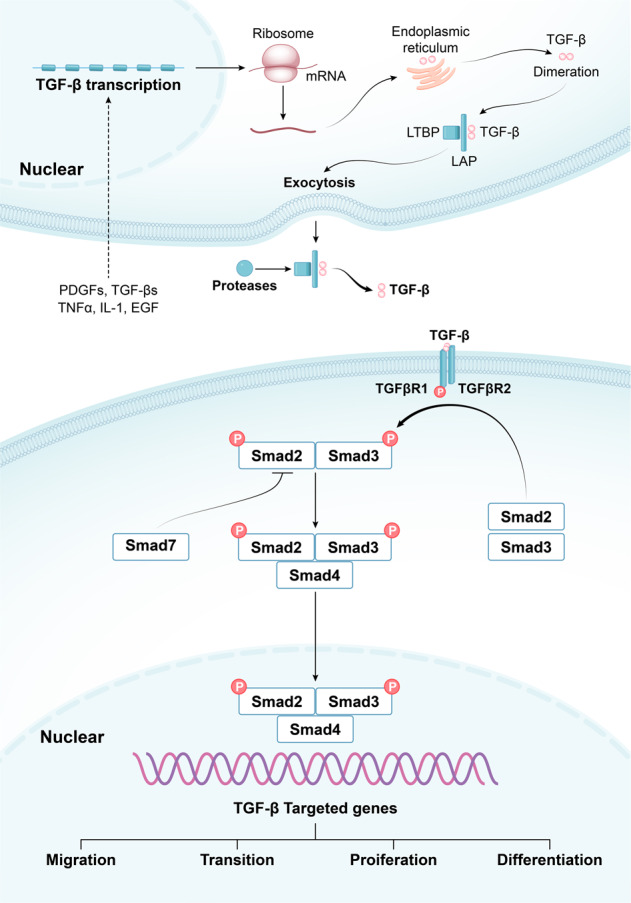
Overview of canonical TGF-β/Smad signaling pathway. Various cytokines stimulate the transcipiton of TGF-β, such as PDGFs, TGF-βs, TNF-α, IL-1β, and EGF. Pro-TGF-β is synthesized in the ribosome and endoplasmic reticulum. After dimeration, LAP binds to mature TGF-β and attaches to LTBP, entering the intercellular space through exocytosis. Actived TGF-β is released by proteases, and binds to TGFβR2 and TGFβR1. Phosphorylated TGFβR2 phosphorylates TGFβR1. TGFβR1 subsequently triggers the phosphorylation of Smad2/3, which interact with Smad4 and enter the nucleus to activate the expression of target genes. Smad7 is a negative regulator of TGF-β/Smad signaling. LAP, latency-associated peptide; LTBP, latent TGF-β binding protein

TGF-βs can regulate fibrosis via both canonical and non-canonical signaling pathways. Smad proteins are the canonical intracellular effector of TGF-β/TGFβR. Activated TGFβR1 subsequently induces phosphorylation of Smad2 and Smad3, which interact with Smad4 and enter the nucleus to activate the expression of target genes^102^. Smad7 is a negative regulator of TGF‑β/Smad signaling^109^ (Fig. 5). TGF-β could also activate non-canonical (non-Smad) signaling pathways, such as PI3K/AKT, mitogen-activated protein kinase (MAPK) pathways, and JAK/ STAT^110^. Macrophages, epithelial cells, and fibroblasts were the main sources of TGF-β in fibrosis^111,112^. TGF-β promotes fibrosis through diverse mechanisms, including activation of resident fibroblasts, promotion of cell apoptosis, and induction of EMT.

##### Fibroblast activation induced by TGF-β

Activated TGF-β1/Smad3 signaling pathway promoted the recruitment of fibroblasts to injury sites and mediated fibroblast-to-myofibroblast differentiation, thus stimulating the secretion of ECM components^113–115^. Reactive oxygen species (ROS) has been reported to mediate TGF-β-induced activation of fibroblasts. NADPH oxidase (Nox) enzymes are important mediators of electron transport from NADPH to oxygen to form ROS^116^. Once produced, ROS could induce the activation of TGF-β1. Nox4 is a member of Nox enzyme family and its expression could be induced by TGF-β in a variety of cells^117^. TGF-β1 treatment increased the level of Nox4 and alpha-smooth muscle actin (α-SMA), a myofibroblast marker, in primary human cardiac fibroblasts, whereas depletion of Nox4 decreased TGF-β1-stimulated α-SMA expression, indicating that ROS mediated TGF-β1-induced activation of cardiac fibroblasts to myofibroblasts^118^. Recent studies have suggested that TGF-β1-driven activation of fibroblasts might involve metabolic reprogramming in fibroblasts and enhancement of glycolytic pathways^119^.

##### Cell apoptosis induced by TGF-β

TGF-β1-induced apoptosis is important in various fibrosis and the mechanisms might differ between different cell types. ROS plays a key role in endothelial cell apoptosis induced by TGF-β. TGF-β1 caused ROS-dependent p38 activation, while p38 inhibition decrased TGF-β1-induced apoptosis^120^. TGF-β1 could also induce apoptosis of mesangial cells in kidney via p53 phosphorylation and Bcl-2 Associated protein X (Bax) up-regulation^121^.

##### EMT regulated by TGF-β

In fibrosis, the most common type of EMT is the type 2 EMT process. Type 2 EMT, mainly caused by inflammation, is closely related to tissue damage repair response and increases myofibroblasts population^122^. TGF-β is a crucial mediator in regulating type 2 EMT process in fibrosis and its interaction with various signals regulates the occurrence of EMT. Oxidative stress induced by TGF-β is an important event in the EMT process. TGF-β increased the level of ROS by upregulating the expression of Nox4, and then activated ERK and mTOR signaling molecules to promote EMT and fibrosis^123^. PI3K/AKT signals also mediated TGF-β-induced EMT^124^.

#### PDGFs/PDGFRs

PDGFs are stimulators of cell division that are required for cell growth and proliferation. They are disulfide-bonded homodimers and heterodimers composed of five different polypeptide chains (subunits), termed AA, AB, BB, CC, and DD^125^. PDGF ligands bind to PDGFRαα, PDGFRαβ and PDGFRββ^126^. PDGF-A and -C subunits mainly bind to the α chain, B subunit to both α and β chains, and D subunit to the β chain only^127^. Upon ligand binding, PDGFRs phosphorylate and activate downstream signals (RAS/MAPK, PI3K/AKT, and JAK/STAT pathways)^128^.

PDGFs are increased in fibrosis. Macrophages, endothelial cells, and fibroblasts have been identified as the main sources of PDGFs^129–132^. Both PDGF-B and PDGF-D were potent factors for HSCs proliferation and migration, therefore potentiating extracellular matrix deposition in liver fibrogenesis^133,134^, which could be mediated by PGDFRβ^135^. However, deficiency of PDGF-C failed to inhibit liver fibrosis or functional liver impairment^136^, but alleviated kidney fibrotic changes in experimental murine kidney fibrosis^137^. In addition to kidney and liver, studies demonstrated that PDGFs contributed to the formation of heart and lung fibrosis via stimulating activation of fibroblasts^138–140^.

#### FGFs/FGFRs

There are 18 members of the FGF superfamily, which are divided into 6 groups according to sequence homology and differences in biological properties: aFGF and bFGF; INT2, KGF, FGF10, and FGF22; FGF4, FGF5, and FGF6; FGF8, FGF17, and FGF18; FGF9, FGF16, and FGF20; FGF19, FGF21, and FGF23^141^. FGF receptors (FGFR1-FGFR4) are mainly composed of a transmembrane domain, a cytoplasmic tyrosine kinase domain, and an extracellular immunoglobulin domain (D1-D3)^142^. FGFs induce the dimerization, activation, and autophosphorylation of FGFRs and activate the RAS-extracellular signal-regulated kinase (ERK), PI3K-AKT, and JAK/STAT pathways^143–145^. The role of FGFs family in liver fibrosis is not clear. FGF19 deficiency protected mice from liver fibrosis progress in animal models^146^. However, direct stimulation of FGF19 decreased pro-fibrotic and pro-inflammatory cytokines expression on HSCs^147^. FGF21 has attracted much attention due to its important role in liver lipid metabolism^148,149^. FGF21 acts in an endocrine, paracrine, and autocrine-like manner via FGFR1-3/β-Klotho (KLB)^150^. FGF21-knockout mice decreased β oxidation and increased the level of free fatty acids in mice fed methionine- and choline-deficient (MCD) diets, promoting lipotoxicity and steatosis^151^. Increasing expression of FGF21 inhibited inflammation in NASH, and synergistically alleviated obesity and insulin resistance^151,152^. For pulmonary fibrosis, the FGF family is a therapeutic target that promotes fibroblast proliferation and migration but inhibits myofibroblast differentiation^153–156^. Inhibition of FGF/FGFR signaling has achieved reduction of pulmonary fibrosis in IPF^157^.

#### VEGFs/VEGFRs

The VEGF family has 6 members: VEGF-A, -B, -C, -D, -E, and placental growth factor (PIGF)^158^. VEGFs, which are similar to PDGF family proteins in structure, regulates vasculogenesis, angiogenesis and immunity^159^. VEGF-A is widely studied in regulating angiogenesis during homeostasis and disease^160^. VEGF-A exerts its biological functions by binding to VEGFR1 and VEGFR2^160^. VEGF-A were decreased in IPF patients, and lung-specific overexpression of VEGF-A attenuated the lung injury and fibrosis in lung fibrosis mouse model^161^. However, studies have shown the important role of VEGF in promoting pulmonary fibrosis^162,163^. The selective splicing of exons contributes to the existence of various subtypes of VEGF-A, including VEGF-A_121_, VEGF-A_165_, VEGF-A_189_, and VEGF-A_206_, among which VEGF-A_165_ is the most abundant isoform in normal tissues^164,165^. Most studies on the role of VEGF-A in fibrosis have not clearly identified the subtype of VEGF-A, and the dual role of VEGF-A in fibrosis might be related to its different subtypes^166^.

#### CTGF signaling pathway

CTGF is a secreted peptide and has been considered as a novel PDGF-related growth factor regulating the proliferation and chemotaxis of fibroblasts^167^. CTGF can combine with other molecules to promote their pro-fibrotic effects, thereby promoting fibrosis. The binding of CTGF with FGFR2 enhanced the binding of FGFR to FGF2 and FGF4, thus activating ERK signaling and promoting proliferation^168^. Additional studies have shown that CTGF could bind to TGF-β1^169^ and was required for the pro-fibrotic activity of TGF-β1^170,171^. TGF-β-induced endogenous CTGF leads to transcriptional repression of Smad7 via inducing the transcription factor TIEG-1, and by this mechanism, CTGF blocks the inhibitory effect of Smad7, resulting in persistent activation of TGF-β signaling^172^.

#### PI3K/AKT

PI3Ks can be activated by receptor-coupled tyrosine kinase activity, small RAS-related GTPases, and heterotrimeric G proteins^173^. The common downstream of receptor-mediated PI3K activation is AKT, which can phosphorylate many substrates related to cell proliferation, autophagy, and motility^173^. Activated PI3K/AKT negatively regulates the activity of mammalian target of rapamycin (mTOR)^174^. The PI3K/AKT/mTOR is a pivotal signaling involved in cell proliferation and differentiation^175^, and was activated in fibrotic foci^176,177^. The activated PI3K/AKT participated in the TGF-β-induced myofibroblasts activation^178^. PI3K/AKT could also regulate angiogenesis by increasing VEGF/VEGFR signaling^179^ and enhanced VEGFA/VEGFR2 signaling in liver fibrosis and angiogenesis^180,181^.

#### JAK/STAT

The JAKs has four members, JAK1, 2, 3, and TYK2^182^. Upon ligand binding, JAKs are activated and subsequently phosphorylate downstream signaling molecules, such as STAT, which in turn migrates to the nucleus regulating targeted gene expression^183,184^. STAT has seven subtypes: STAT1, 2, 3, 4, 5 A, 5B, and 6^185,186^. JAK signal-mediated transduction depends on the activation of PI3K/AKT/mTOR signaling^187,188^. Inhibition of PI3K/AKT/mTOR enhanced the effect of JAK2 inhibitors on primary human myeloproliferative neoplasm cells^189^. JAK/STAT could also be regulated by PDGFs. JAK2 and STAT3 was upregulated in left atrial and left ventricular fibroblasts treated with PDGF-AB^190^. Inhibition of JAK2 and STAT3 reversed PDGF-AB-induced collagen production in fibroblasts, suggesting that JAK2/STAT3 signaling was involved in PDGF-AB-induced fibrosis^190^. Furthermore, the activation of JAK/STAT signaling is required for TGF-β-mediated CTGF production in primary mouse HSCs^191^. JAK/STAT signals together with TGF-β1/Smad signals promote the EMT process in liver fibrosis^192^.

#### WNT/β-catenin

β-catenin is a transcription factor and its expression is mainly regulated by WNT proteins^193,194^. WNT/β-catenin activate and synergize with TGF-β1 to mediate the activation of myofibroblasts in lung fibrosis^195,196^. WNT/β-catenin signal was upregulated in TGF-β stimulated human lung fibroblasts^197,198^. Blocking β-catenin induced by TGF-β in vivo and in vitro can alleviate BLM-induced lung fibrosis^199^. In liver fibrosis, WNT/β-catenin also regulated the vimentin, collagen 1, and fibronectin in HSCs induced by TGF-β^200^. Apart from TGF-β, WNT/β-catenin can be regulated by CTGF via binding to the WNT coreceptor LDL receptor-related protein 6 (LRP6)^201^.

#### Apoptosis signal-regulating kinase 1 (ASK1) signaling pathway

ASK1 is involved in regulating glucose metabolism and maintaining energy homeostasis, which could activate the p38/cJun NH2-terminal kinase (JNK) signaling pathway^202^. Activation of the JNK signaling cascade suppressed the PPARα and FGF21 pathways^203^. Inhibition of ASK1 reduced insulin resistance, hepatic steatosis, inflammation, and fibrosis^204,205^.

### Regulation of ECM cross-linking

Lysyl oxidases (LOXs) catalyses the conversion of lysine molecules to highly reactive aldehydes and enhances ECM (primarily collagen and elastin) cross-linking^206,207^. LOX family includes lysyl oxidase (LOX) and four lysyl oxidase-like proteins (LOXL1-4)^208–210^. The interaction of LOXs with TGF-β mediates the pro-fibrotic effect of LOXs in fibrosis. LOXL1 was required for TGF-β1 induced HSCs activation in liver fibrosis^211^. LOXL1 deficiency protected against TGF-β1-activated fibrosis and decreased the expression of fibrotic genes in vivo^212^. Silencing LOXL2 decreased mouse lung fibroblast proliferation and the levels of collagen 1α1 (COL1A1) via inhibition of TGF-β1/Smad2/3^213^.

### Regulation of metabolism and inflammation

**Fig. 6:**
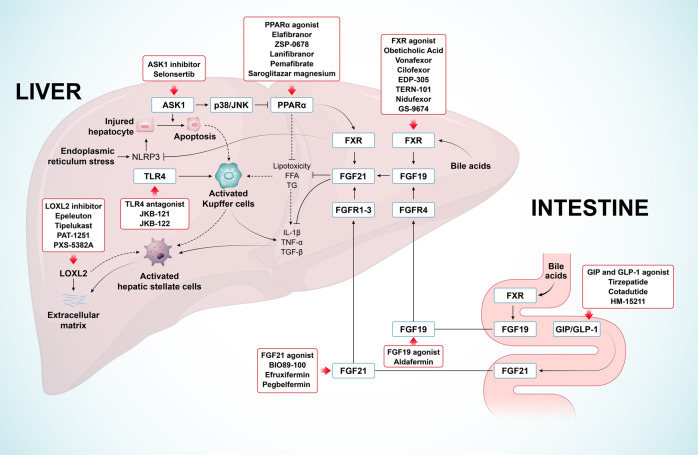
Molecular signaling pathways of NASH and a summary of related target drugs. FFA, free fatty acid; TG, triglycerides

Alterations in metabolism can regulate the activation of inflammation-related pathways in epithelial cells, immune cells, and fibroblasts. The interactions between metabolism- and inflammation-related pathways modulate myofibroblasts activation. Signaling molecules that regulate metabolism may provide an interesting avenue for slowing the progression of fibrosis. As most of these signaling pathways regulating metabolism and inflammation are essential for NASH develpoment, the interactions between these signaling pathways in NASH are shown in Fig. 6.

#### Peroxisome proliferator-activated receptors (PPARs) signaling pathway

PPARs are the nuclear receptors dependent on ligand binding^214^ and activate targeted genes related to lipid and glucose metabolism and adipogenesis^215,216^. There are three PPARs: PPARα, PPARγ, and PPARβ (also called δ)^217,218^. PPARα is most expressed in brown adipose tissue and liver^219^. The correlation of PPARs with liver fibrosis, especially NASH, is well-elaborated. PPARα is important for fatty acid metabolism^220^. Increased oxidative stress and hepatocyte apoptosis with higher NASH scores were observed in Pparα-null mice fed a high-fat diet^221^. Treatment with PPARα ligands attenuated liver fibrosis in rat thioacetamide models of liver cirrhosis^222^. Fasting-induced PPARα^−/−^ mice showed low levels of FGF21, whereas FGF21 reduced hepatic triglycerides and cholesterol esters only in WT mice, suggesting that the effect of FGF21 on lipid metabolism might be partially dependent on PPARα^223^.

The function of PPARγ in NASH is more dependent on its role in inflammation. PPARγ activation inhibited inflammatory responses by inactivating nuclear factor-κB (NF-κB) signaling^224^ and reducing TNF-α and IL-1β expression in monocytes and macrophages^225^. Dual activation of PPARγ and PPARα has a favourable effect in ameliorating NASH by reducing inflammation, steatosis, and fibrosis^226,227^. PPAR-α and PPAR-γ activators have achieved efficacy in cardiac fibrosis^228^, renal fibrosis^229^ and pulmonary fibrosis^230^ animal models.

PPARβ/δ is mainly expressed in hepatocytes, Kupffer cells, and HSCs in liver^231,232^. PPARβ/δ-null mice exhibited aggravated hepatoxicity in carbon tetrachloride (CCl_4_)-treated mice^233^. However, the contraditory effects of PPARβ/δ agonists on HSCs proliferation and liver fibrosis hindered PPARβ/δ agonists from entering clinical trials^234–236^, which might be due to discrepancies in the ligands, dosage, and in vivo pharmacological properties of compounds.

#### Farnesoid X receptor (FXR) signaling pathway

FXR, as a nuclear receptor mainly located in enterohepatic tissues, can be activated by bile acids and regulate lipid and glucose metabolism^237–239^. FXR forms a heterodimer with the 9-cis-retinoic acid receptor and binds to farnesoid X response elements (FXREs), thus regulating target gene expression^240^. The roles of FXR vary in different organs. FXR expression was upregulated in lung fibrosis, and inhibition of FXR inhibited the bile acid-induced EMT and activation of lung fibroblasts^241^. However, FXR was reported to exert anti-fibrotic effect on kidney fibrosis and liver fibrosis. Treatment with FXR-activating ligand ameliorated triglyceride accumulation, improved proteinuria, and decreased ECM deposition in kidney disease experimental models^242^. FXR activation also protected hepatocytes from liver injury by inhibiting the activation of the NACHT, LRR, and PYD domain-containing protein 3 (NLRP3) inflammasome^243^. The interaction of FXR with other molecules is involved in bile acids circulation and plays an important role in NASH^244^. PPARα activation was required for the mRNA expression of FXR in the liver of fasted mice^245^. FXR directly regulated the expression of FGF19, thereby regulating hepatic protein and glycogen metabolism^246,247^. FXR/FGF19 axis increased FGF21 secretion^248,249^. FXR might also directly activate the expression of FGF21 by interacting with the FXRE in the 5’-flanking region of the FGF21 gene^248^.

#### Toll-like receptor 4 (TLR4) signaling pathway

TLR4, a member of the TLR family, functions as a crucial regulator in the immune system and inflammatory response. Fibroblast-specific deletion of TLR4 protected from mice lung and skin fibrosis^250^. In liver fibrosis, HSCs are the main effector cells of TLR4. TLR4 could sensitize HSCs to TGF-β stimulation and promote the activation of Kupffer cells, regulating hepatitis and liver fibrosis^251^. Activation of the TLR4/NF-κB signaling pathway induced hepatic inflammation^252,253^. However, TLR4 is an important receptor for AT2 proliferation and deletion of TLR4 in surfactant-protein-C-positive AT2 cells leads to impaired renewal capacity, severe fibrosis and mortality in IPF^254^.

#### GIP/GIPR and GLP-1/GLP-1R

Gastric inhibitory polypeptide (GIP) and glucagon-like peptide-1 (GLP-1) are the two major incretin hormones produced by the intestine that regulate insulin and glucagon secretion and food ingestion^255^. GIP is secreted by K cells in the upper part of the small intestine, while GLP-1 is mainly released by intestinal endocrine cells^256–258^. GIP exerts biological functions via binding to its receptor GIPR^259,260^ and was related to the activation of macrophages^261,262^. GLP-1 is expressed in various cells and binds to GLP-1R^263^. GLP-1 could downregulate collagen expression and TGF-β1 expression via regulating FGF21 in NASH mouse models^264,265^ and activating AMP-activated protein kinase (AMPK) in diabetic lung fibrosis^266^. Combined treatment with GLP-1R and GIPR agonists improved NASH steatosis, lobular inflammation, hepatocyte ballooning, and fibrosis^267^.

## Anti-fibrotic drugs and clinical trials

Numerous small molecules or compounds are currently in clinical trials for fibrosis. Published clinical data on these compounds were listed in Table 1, and we categorized these drugs by targets and then ranked each target drug by clinical trial grade (marketed, phase 3, phase 2, and phase 1). Accordingly, antifibrotic drugs that have published clinical data and are in Phase 2, Phase 3 clinical trials or marketed are summarized in this part based on the ranking results.

**Table 1:** Drug targets and NCT number of clinical trials

Target		Drug Name	Conditions	Highest Status (phase)	NCT	Status	Sample size
TGF-β/TGFβR	TGF-βs	Pirfendione	IPF	Marketed	NCT00662038	Completed	1058
	p38 MAPK, TGFβ1, FGFR1	Hydronidone	Liver fibrosis	III	NCT05115942	Recruiting	248
	TGFβR1	HEC-585	IPF	II	NCT05060822	Recruiting	270
	αV/β1, αV/β6	PLN-74809	IPF	II	NCT04396756	Recruiting	112
	αV/β6, TGF-β	BG00011	IPF	II	NCT03573505	Terminated	109
	αV/β1, αV/β3, αV/β6	IDL-2965	IPF	I	NCT03949530	Terminated	6
	TGF-β1	TRK-250	IPF	I	NCT03727802	Completed	34
	TGF-βs	Luspatercept	Myelofibrosis	III	NCT04717414	Recruiting	309
	TGF-β, BMPRII	Sotatercept	Myelofibrosis	II	NCT01712308	Completed	63
	TGF-β1 and TGF-β3	AVID200	Myelofibrosis	I	NCT03895112	Active, not recruiting	22
FGF	FGF21	BIO89-100	NASH	II	NCT04048135	Active, not recruiting	101
	FGF21	Efruxifermin	NASH	II	NCT03976401	Completed	110
	FGF21	Pegbelfermin	NASH	II	NCT02413372	Completed	184
	FGF19	Aldafermin	NASH	II	NCT03912532	Completed	171
RTKs	PDGFRs, FGFRs, VEGFRs	Nintedanib	IPF	Marketed	NCT02598193	Completed	89
	PDGFRα, β, FGFR1-4, and VEGFR1-3	ZSP1603	IPF	II	NCT05119972	Recruiting	36
	β-Klotho/FGFR1c receptor complex	MK-3655	NASH	II	NCT04583423	Recruiting	328
CTGF	CTGF	Pamrevlumab	IPF	III	NCT03955146	Recruiting	340
PI3K	PI3Kδ	Parsaclisib	Myelofibrosis	III	NCT04551053	Recruiting	212
	PI3K/mTOR	Omipalisib	IPF	I	NCT01725139	Completed	17
	PI3K/mTOR	HEC-68498	IPF	I	NCT03502902	Completed	55
	PI3K p110α/β/δ/γ	Buparlisib	Myelofibrosis	I	NCT01730248	Terminated	63
	PI3Kδ, CK1-epsilon	Umbralisib	Myelofibrosis	I	NCT02493530	Active, not recruiting	60
JAK	JAK1/2	Ruxolitinib	Myelofibrosis	Marketed	NCT02386800	Recruiting	356
	JAK2, FLT3	Fedratinib	Myelofibrosis	III	NCT03755518	Active, not recruiting	110
	JAK1/2, TBK1, ACVR1/ALK2	Momelotinib	Myelofibrosis	III	NCT04173494	Active, not recruiting	195
	JAK2, FLT3, IRAK1	Pacritinib	Myelofibrosis	III	NCT03165734	Recruiting	348
	JAK1/2/3	Jaktinib	Myelofibrosis	III	NCT04617028	Recruiting	105
	JAK1	Itacitinib	Myelofibrosis	II	NCT04640025	Recruiting	100
	JAK2	Ilginatinib	Myelofibrosis	II	NCT01423851	Completed	77
WNT/β-catenin	WNT	SM04646	IPF	II	NCT03591926	Withdrawn	0
	β-catenin	PRI-724	liver cirrhosis	II	NCT03620474	Completed	27
ASK, MAPK	ASK1, MAPKKK5	Selonsertib	NASH	III	NCT03053050	Terminated	808
	JNK1, MAPK8	CC-90001	NASH	II	NCT04048876	Terminated	56
	MAP3K19	MG-S-2525	IPF	I	NCT03650075	Completed	81
LOXL	LOXL2, LTD4 receptor, PDE3 /4	Epeleuton	NAFLD	II	NCT02941549	Completed	96
	LOXL2, LTD4 receptor, PDE3 /4	Tipelukast	IPF	II	NCT02503657	Completed	15
	LOXL2	PAT-1251	Myelofibrosis	II	NCT04054245	Withdrawn	0
	LOXL2	PXS-5382A	IPF, NASH	I	NCT04183517	Completed	18
PPAR	PPAR α/δ	Elafibranor	NASH	III	NCT02704403	Terminated	2157
	PPAR α/γ	Saroglitazar	NASH	III	NCT04193982	Recruiting	250
	PPAR α/δ/γ	Lanifibranor	NASH	III	NCT04849728	Recruiting	2000
	PPAR α	Pemafibrate	NASH	II	NCT03350165	Completed	118
	PPARα/δ	ZSP0678	NASH	I	NCT04137055	Completed	104
FXR	FXR	Obeticholic Acid	NASH	III	NCT02548351	Active, not recruiting	2480
	FXR	Cilofexor	Liver fibrosis, NASH	II	NCT02854605	Completed	140
	FXR	Nidufexor	NASH	II	NCT02913105	Terminated	122
	FXR	TERN-101	NASH	II	NCT04328077	Completed	101
	FXR	Vonafexor	NASH	II	NCT03812029	Completed	120
	FXR	EDP-305	NASH	II	NCT04378010	Recruiting	336
	FXR	Tropifexor	NASH	II	NCT04147195	Terminated	41
TLR	TLR4	JKB-121	NASH	II	NCT02442687	Completed	65
	TLR4	JKB-122	NASH	II	NCT04255069	Active, not recruiting	300
GLP/GIP	GLP-1 receptor	Semaglutide	NASH	III	NCT04822181	Recruiting	1200
	GLP-1/GIP receptor	Tirzepatide	NASH	II	NCT04166773	Recruiting	196
	GLP-1/Glucagon receptor	Cotadutide	NASH	II	NCT05364931	Active, not recruiting	1860
	GLP-1/GIP/Glucagon	HM-15211	NASH	II	NCT04505436	Recruiting	217
CFTR	CFTR	Elexacaftor	Cystic fibrosis	III	NCT03525444	Completed	405
	CFTR	Ivacaftor	Cystic fibrosis	III	NCT01707290	Completed	125
	CFTR	GLPG1837	Cystic fibrosis	II	NCT02707562	Completed	26
	CFTR	FDL169	Cystic fibrosis	II	NCT02767297	Completed	46
	CFTR	Olacaftor	Cystic fibrosis	II	NCT02951182	Completed	74
	CFTR	VX-152	Cystic fibrosis	II	NCT02951195	Completed	80
	CFTR	MRT5005	Cystic fibrosis	II	NCT03375047	Recruiting	40
	CFTR	GLPG2737	Cystic fibrosis	II	NCT03474042	Completed	22
	CFTR	Nesolicaftor	Cystic fibrosis	II	NCT03591094	Completed	40
	CFTR	VX-121	Cystic fibrosis	II	NCT03912233	Completed	87
	CFTR	ABBV-3067	Cystic fibrosis	II	NCT03969888	Active, not recruiting	189
	CFTR	ELX-02	Cystic fibrosis	II	NCT04135495	Recruiting	16
	CFTR	Eluforsen	Cystic fibrosis	II	NCT02532764	Completed	70
	CFTR	Dirocaftor	Cystic fibrosis	II	NCT03251092	Completed	179
	CFTR	FDL176	Cystic fibrosis	I	NCT03173573	Completed	109
	CFTR	Posenacaftor	Cystic fibrosis	I	NCT03140527	Completed	171
	CFTR	GLPG2451	Cystic fibrosis	I	NCT02788721	Completed	31
HDAC	HDAC	Panobinostat	Myelofibrosis	Marketed	NCT02386800	Recruiting	356
	HDAC	Pracinostat	Myelofibrosis	II	NCT01200498	Completed	23
THRβ	THRβ	Resmetirom	NASH	III	NCT03900429	Recruiting	2000
	THRβ	VK2809	NASH	II	NCT04173065	Recruiting	337
CCR	CCR2/CCR5	Cenicriviroc	NASH	III	NCT03028740	Terminated	1778
Galectin	Galectin-3	Belapectin	NASH	III	NCT04365868	Recruiting	1010
	Galectin-3	GB1211	NASH	II	NCT04607655	withdrawn	0
	Galectin-3	GB0139	IPF	II	NCT03832946	Active, not recruiting	426
MPC	MPC	Azemiglitazone potassium	NASH	III	NCT03970031	Active, not recruiting	1800
	MPC	Deuterium-Stabilized (R)-Pioglitazone	NASH	II	NCT04321343	Active, not recruiting	123
SCD	SCD-1	Aramchol	NASH	III	NCT04104321	Recruiting	2000
ATX	ATX	Ziritaxestat	IPF	III	NCT03711162	Terminated	526
FATP5	FATP5	Ursodiol	Cystic Fibrosis	II	NCT00004315	Unkonwn	20
ACC	ACC1/2	PF-05221304	NASH	II	NCT03248882	Completed	305
	ACC	Firsocostat	NASH	II	NCT03449446	Completed	395
PDE	PDEs (mainly PDE2)	ZSP1601	NASH	II	NCT04140123	Completed	37
	LOXL2, LTD4 receptor, PDE3 /4	Epeleuton	NAFLD	II	NCT02941549	Completed	96
	LOXL2, LTD4 receptor, PDE3 /4	Tipelukast	IPF	II	NCT02503657	Completed	15
	PDE 3/4	Ensifentrine	Cystic fibrosis	II	NCT02919995	Completed	10
AMPK	AMPK	PXL-770	NAFLD	II	NCT03763877	Completed	121
MMP	MMP2, MMP9, VEGF-A	ALS-L1023	NASH	II	NCT04342793	Unknown	60
A3AR	A3AR	Namodenoson	NASH	II	NCT02927314	Completed	60
FASN	FASN	TVB-2640	NASH	II	NCT03938246	Completed	142
Bioidentical testosterone	Bioidentical testosterone	LPCN 1144	NASH	II	NCT04134091	Completed	56
Stem cell	Stem cell	HepaStem	NASH	II	NCT03963921	Completed	23
HSP	HSP 47	BMS-986263	NASH	II	NCT04267393	Recruiting	270
	HSP 90	PU-H71	Myelofibrosis	I	NCT03935555	Recruiting	24
CD	CD3	Foralumab	NASH	II	NCT03291249	Withdrawn	0
	CD123	Tagraxofusp	Myelofibrosis	II	NCT02268253	Recruiting	130
ileal bile acid transport	ileal bile acid transport	Elobixibat	NASH	II	NCT04006145	Completed	47
aldosterone receptor	aldosterone receptor	Apararenone	NASH	II	NCT02923154	Completed	48
GPR	GPR-35	RVT1601	IPF	II	NCT03864328	Terminated	108
	GPR-84	GLPG-1205	IPF	II	NCT03725852	Completed	68
	GPR-40, GPR-84	PBI-4050	IPF	II	NCT02538536	Completed	41
ROCK2	ROCK2	Belumosudil	IPF	II	NCT02688647	Completed	76
BAFFR	BAFFR	Ianalumab	IPF	II	NCT03287414	Terminated	30
LPA1	LPA1	BMS-986278	IPF	II	NCT04308681	Recruiting	360
Telomerase	Telomerase	Imetelstat	Myelofibrosis	III	NCT04576156	Recruiting	320
KHK	KHK	PF-06835919	NASH	II	NCT03969719	Completed	164
calpain	calpain 1, 2, and 9	BLD-2660	IPF	II	NCT04244825	Withdrawn	0
P selectin	P selectin	Crizanlizumab	Myelofibrosis	II	NCT04097821	Recruiting	243
SMO	SMO	Sonidegib	Myelofibrosis	II	NCT01787552	Completed	50
Bcl-2	Bcl-2	Navitoclax	Myelofibrosis	II	NCT03222609	Active, not recruiting	191
BET family	BET family	Pelabresib	Myelofibrosis	II	NCT02158858	Recruiting	341
ENaC	ENaC	BI-1265162	Cystic fibrosis	II	NCT04059094	Terminated	52
	ENaC	P-1037	Cystic fibrosis	II	NCT02343445	Completed	142
	ENaC	QBW276	Cystic fibrosis	II	NCT02566044	Completed	16
	ENaC	IONIS-ENaCRx	Cystic fibrosis	I	NCT03647228	Completed	98
	ENaC	AZD5634	Cystic fibrosis	I	NCT02950805	Completed	9
	ENaC	BI 443651	Cystic fibrosis	I	NCT02976519	Completed	64
	ENaC	Idelalisib	Myelofibrosis	I	NCT02436135	Terminated	10
DNase I	DNase I	AIR DNase	Cystic fibrosis	II	NCT02722122	Unkonwn	15
AA/DHA imbalance	AA/DHA imbalance	Fenretinide	Cystic fibrosis	II	NCT03265288	Completed	166
Neutrophil elastase	Neutrophil Elastase	Lonodelestat	Cystic fibrosis	II	NCT03748199	Completed	32
	Neutrophil Elastase	CHF 6333	Cystic fibrosis	I	NCT04010799	Completed	68
leukotriene B4	leukotriene B4	Acebilustat	Cystic fibrosis	II	NCT02443688	Completed	200
CDK	CDK1, CDK2/E, CDK2/A, CDK5, 7, 9	Seliciclib	Cystic fibrosis	II	NCT02649751	Terminated	49
	CDK4/6	Ribociclib	Myelofibrosis	I	NCT02370706	Completed	15
LSD	LSD1	Bomedemstat bis-tosylate	Myelofibrosis	II	NCT03136185	Completed	89
MDM2	MDM2	KRT-232	Myelofibrosis	III	NCT03662126	Recruiting	385
PLK1	PLK1	Rigosertib	Myelofibrosis	II	NCT02730884	Terminated	3
IL-1α	IL-1α	Bermekimab(MABp1)	Systemic Sclerosis	II	NCT04045743	Active, not recruiting	20
HSD17B13	HSD17B13	ARO-HSD	NASH	I	NCT04202354	Completed	50
MOTS-c	MOTS-c	CB4211	NAFLD	I	NCT03998514	Completed	88
IFN-γ	IFN-γ	Interferon gamma	IPF	I	NCT00563212	Completed	12
Autotaxin	Autotaxin	BBT-877	IPF	I	NCT03830125	Completed	88
Glutathione dependent PGD synthase	Glutathione dependent PGD synthase	ZL-2102	IPF	I	NCT02397005	Unknown	120
Arginase	Arginase	CB-280	Cystic fibrosis	I	NCT04279769	Completed	32
GSNOR	GSNOR	N-6022	Cystic fibrosis	I	NCT01746784	Completed	66
Pim kinase inhibitor	Pim-1, -2, -3 kinase	TP-3654	Myelofibrosis	II	NCT04176198	Recruiting	60
PRMT	PRMT5	PRT-543	Myelofibrosis	I	NCT03886831	Active, not recruiting	227

*AA/DHA* ascorbic acid/ docosahexaenoic acid, *ACC* acetyl-coenzyme A carboxylase, *ACVR1* activin A receptor type 1, *ALK2* activin receptor-like kinase 2, *ATX* autotoxin, *A3AR* A3 adenosine receptor, *BET family* bromodomain and extra-terminal domain family, *BMPRII* bone morphogenic protein receptor type II, *CCR2* chemokine receptor 2, *CCR5* chemokine receptor 5, *CDK* cyclin-dependent kinase, *EnaC* epithelial sodium channel, *FASN* fatty acid synthase, *FATP5* fatty acid transport protein 5, *FLT3* FMS-like tyrosine kinase 3, *GSNOR* S-nitrosoglutathione reductase, *HDAC* histone deacetylase, *HSD17B13* 17-beta hydroxysteroid dehydrogenase 13, *HSP47* heat shock protein 47, *IRAK1* Interleukin-1 receptor-associated kinases, *KHK* ketohexokinase, *LSD1* lysine-specific demethylase 1, *LTD4* leukotriene D4, *MAPKKK5* MEK Kinase5, *MDM2* mouse double minute 2, *MMP* matrix metallopeptidase, *MOTS-c* mitochondrial open reading frame of the 12S rRNA-c, *MPC* mitochondrial pyruvate carrier, *NASH* non-alcoholic steatosis, *PDE* phosphodiesterase, *PLK1* polo-like kinase 1, *PRMT5* protein arginine methyltransferase 5, *TBK1* TANK-binding kinase 1, *THRβ* thyroid hormone receptor beta, *SCD-1* stearoyl CoA desaturase-1, *SMO* Smoothened, *SP-B* surfactant proteins B

### Anti-fibrotic drugs targeting TGF-β

Most anti-TGF-β therapeutic drugs fall into five groups^268,269^: (1) nucleic acid drugs that blocking TGF-β synthesis. (2) TGF-β receptor kinases inhibitors, which block ATP binding to TGFβR, thus inhibiting Smad2 and Smad3 activation. (3) monoclonal antibodies preventing TGF-β from binding to its receptors. (4) high-affinity ligand traps prevent TGF-β from binding to its receptor. These inhibitors contain TβRII extracellular domains that could prevent TGF-β1 and TGF-β3 binding to TβRII receptors. (5) Some antibodies or molecules inhibiting the TGF-β activation, for example, drugs targeting αv/β integrins. Anti-fibrotic drugs targeting TGF-β now in clinical trials are mainly used in two diseases, IPF and myelofibrosis. Selected drugs targeting TGF-βs are described in detail.

### Pirfenidone

Pirfenidone (PFD) is one of two FDA-approved drugs for IPF^270^, which inhibits both the synthesis and activition of TGF-βs^271^. The action mechanism of PFD in IPF has not been fully elaborated. Studies showed that PFD could inhibit the fibroblasts activation, reducing the synthesis of type 1 and type 3 collagen and the deposition of ECM^272–275^. Clinical trials demonstrated that PFD reduced lung function decline, decreased mortality, and improved overall survival of IPF patients^276–280^. Anorexia, rash, and gastrointestinal disorders are reported to be common side effects of PFD^281^. Based on the effect of PFD on improving inflammation and fibrosis in IPF, clinical studies on PFD for other types of pulmonary fibrosis are in progress. HEC-585 is a pyrimidine compound that is structurally related to PFD. Two phase I clinical trials were carried out to evaluate the safety, tolerability, and pharmacokinetics of HEC-585 in healthy subjects (NCT04512170 and NCT03092102).

### Hydronidone

Hydronidone is a derivative of PFD with potential therapeutic efficacy for hepatic fibrosis^282^. The results of an open-label, randomized, dose-escalating study showed that hydronidone was well tolerated and effectively absorbed in healthy Chinese subjects (ChiCTR-ONC-12002899)^282^. Currently, a phase III study on the efficacy of hydronidone in HBV-induced liver fibrosis is in progress.

### Luspatercept

Luspatercept is a recombinant fusion protein that binds TGF-β ligands to reduce Smad2/3 signaling. Luspatercept has been evaluated in myelofibrosis-associated anemia with 33 patients received concomitant ruxolitinib. Among transfusion-independent patients, 2 patients who did not receive ruxolitinib (10%) and 3 patients who received ruxolitinib (21%) experienced an increase of hemoglobin about 1.5 g/dL over 12 weeks. In the transfusion dependent cohort, 2 patients who did not receive ruxolitinib and 6 patients who received ruxolitinib were transfusion independent for at least 12 weeks^283^

### AVID-200

AVID-200 contains soluble, dimerized, Fc-linked TβRII ectodomains and can be a high-affinity ligand trap preventing TGF-β from binding to its receptor. Treatment of myelofibrosis mononuclear cells with AVID-200 increased numbers of progenitor cells with wild type JAK2 but not mutated JAK2V617F^284^. Phase 1 clinical study in 12 myelofibrosis patients with ruxolitinib resistant showed that eight patients with grade 3/4 adverse reactions did not have dose-limiting toxicity and had improved platelet counts, with an average increase of 48%^283^.

### Anti-fibrotic drugs targeting RTKs

#### Nintedanib

Nintedanib is a receptor tyrosine kinase inhibitor (RTKs: FGFRs, VEGFRs, and PDGFRs) that targets growth factor pathways, including FGFRs, VEGFRs, and PDGFRs^285^. In BLM-treated and silica-induced fibrosis mouse models, nintedanib reduced lung inflammation and fibrosis by decreasing total collagen, inflammatory chemokines, and pro-fibrotic factors both in therapeutic and preventive regimens^157,286^. Clinical trials have shown that nintedanib decreased the decline in FVC^287^ and reduced disease progression in IPF patients^288–291^. Nintedanib had acceptable safety and tolerability^292,293^, of which nausea and diarrhoea were the common side effects in the treatment of IPF^294^. The combination of PFD and nintedanib might produce synergistic effects and provide new prospects for the treatment of IPF^277^. However, both nintedanib and PFD have some problems such as high liver toxicity, high dosage, and photoallergic reaction, thus their long-term drug tolerance needs to be further determined.

#### ZSP1603

ZSP1603 (also known as WXFL-152), identified from a series of 4-hydroxyquinoline derivatives, targets VEGFR2, FGFRs, and PDGFRβ^295^. Our previous study showed the ability of ZSP1603 to reduce pulmonary injury, inflammation, and fibrosis in BLM-treated mice and rats^296^. ZSP1603 could inhibit the proliferation of primary human pulmonary fibroblasts (pHPFs) by blocking the PDGFRβ/ERK signaling pathway and decrease the differentiation of pHPFs by reducing TGF-β1, tissue inhibitor of metalloproteinase -1, and COL1A1^296^. The clinical study of ZSP1603 is expected to provide a new choice for IPF therapy.

### Anti-fibrotic drugs targeting CTGF

#### Pamrevlumab

Pamrevlumab is a recombinant antibody that targets CTGF and inactivates its downstream inflammatory signals^170^. In a phase II, randomized, double-blind, placebo-controlled PRAISE trial involving 7 countries, pamrevlumab decreased the decline in FVC and inhibited the disease progression of IPF (NCT01890265)^297^. More therapeutic effects of pamrevlumab is expected to be investigated in phase III clinical trials (NCT04419558).

### Anti-fibrotic drugs targeting PI3K

PI3K/AKT palys an important role in fibrotic processes and represents a critical target for the development of novel anti-fibrotic strategies. PI3K/AKT inhibitors are currently in clinical evaluation in IPF and myelofibrosis.

#### Parsaclisib

Parsaclisib is a potent PI3Kδ inhibitor and exerts antitumour effects in models of B-cell malignancy^298^. Single-dose parsaclisib alone or combination with itraconazole or rifampin achieved safety and toleratility in healthy subjects^299^. Two clinical trials in phase III studies (NCT04551066 and NCT04551053) were launched to test the efficacy and safety of parsaclisib and ruxolitinib in myelofibrosis.

#### Omipalisib

Omipalisib (GSK-2126458) is a dual inhibitor of PI3K/mTOR. Omipalisib inhibited the proliferation of pHPFs and decreased collagen accumulation induced by TGF-β1 in pHPFs^176^. Omipalisib was well absorbed and reached the lung in a randomized, placebo-controlled, double-blind phase I study in subjects with IPF (NCT01725139)^300^. Diarrhoea was the most commonly reported side effect of omipalisib^300^.

### Anti-fibrotic drugs targeting JAKs

Since JAKs are essential for the occurrence and development of myelofibrosis, JAK inhibitors have achieved improvements in quality of life in patients with myelofibrosis. However, most drugs targeting JAK/STAT did not seem to prevent myelofibrosis patients from progressing to acute myeloid leukemia^301^.

#### Ruxolitinib

Ruxolitinib, a JAK1/JAK2 inhibitor, is approved by the FDA for patients with intermediate- and high-risk myelofibrosis. The effect of ruxolitinib in anemic myelofibrosis patients was evaluated in a phase 2 study (NCT02966353), who received ruxolitinib at 10 mg for the first 12 weeks, followed by escalating doses to 25 mg. During the study, palpable spleen length was reduced at least 50% in 70% patients receiving ruxolitinib, but 11.8% of patients needed platelet transfusion. The results also showed that the platelet counts and hemoglobin level of patients receiving increased dose were similar to those of patients who did not receice a dose increase^302^.

#### Momelotinib

Momelotinib (also known as CYT387, a JAK1/2 inhibitor) showed favorable therapeutic effects on myelofibrosis in preclinical trials by reducing multiple myeloma proliferation, inducing apoptosis of JAK2-dependent haematopoietic cells, and regulating inflammatory cytokines^303^. In a phase 3 study (NCT02101268), 156 patients with myeloid fibrosis were assigned to receive momelotinib (104) or standard care (52, 89% of whom received ruxolitinib). Encountered with the standard intervention group (6% of patients), 7% of patients in the momelotinib group had at least a 35% reduction in spleen volume. 11% of patients experienced peripheral neuropathy in the momelotinib group, compared with none in the standard intervention group^304^. Moreover, compared with ruxolitinib, the blood transfusion requirements and drug dependence of momelotinib were markedly reduced^305^.

#### Fedratinib

Fedratinib is a JAK2 inhibitor and has been used in treatment for patients with myeloproliferative neoplasm-associated myelofibrosis^306^. After 24 weeks, patients in the 400 mg fedratinib group had a 47% spleen volume response rate compared with 1% of patients with myelofibrosis in the placebo group. In this study, the two most common adverse reactions in patients taking fedratinib were anemia and diarrhea^307^.

#### Pacritinib

Pacritinib is an inhibitor of JAK2 and FMS-like tyrosine kinase 3. Pacritinib has good tolerance and clinical activity in myelofibrosis^308,309^. Twice daily pacritinib resulted in a significant reduction in spleen volume and improvements in the total symptom score over the best available therapy for myelofibrosis^310^.

#### Itacitinib

Itacitinib (INCB039110), a selective JAK1 inhibitor, has demonstrated favourable safety and anticancer effects^311^. Itacitinib exerts its anti-inflammatory effects by reducing pro-inflammatory cytokines and regulating the polarization of macrophages^312^. Administration of itacitinib at 200 mg twice daily and 600 mg once daily reduced the total symptom score in patients with myelofibrosis, and decreased the requirement of red blood cell units transfused in patients who needed transfusions during the 12 weeks prior to itacitinib treatment (NCT01633372)^313^.

### Anti-fibrotic drugs targeting β-catenin

#### PRI-724

PRI-724 (also known as ICG-001) is a small molecule drug that modulate β-catenin/CBP transcription^314,315^. Preclinical studies demonstrated the efficacy of PRI-724 in decreasing ECM deposition and hepatic inflammation in a mouse model of CCl_4_-induced acute liver injury^315^ and a mouse model of HCV-infection^316^. In a dose escalation phase I trial, PRI-724 was well-tolerated in patients with HCV-induced cirrhosis at the dose of 10 or 40 mg/m(2) daily for 12 weeks^317^. However, PRI-724 did not effectively reduce liver fibrosis in patients with HCV- and HBV-induced cirrhosis, either by sequential scoring or by measuring proportional area of collagen for 12 weeks, but significantly improved liver stiffness (NCT03620474)^318^.

### Anti-fibrotic drugs targeting ASK-1

#### Selonsertib

Selonsertib (GS-4997), a small molecule inhibitor of ASK1, showed efficacy in reducing collagen deposition, fibrosis stage, steatosis, and inflammation in a phase 2 study^319^. However, the phase III clinical trial (NCT03053050) of selonsertib was terminated in NASH patients with bridging fibrosis or compensated cirrhosis because its effect in alleviating fibrosis was not obvious^320^.

### Anti-fibrotic drugs targeting PPARs

Since PPARs are involved in glucose and lipid metabolism, PPARs ligands are expected to be promising therapeutic agents for NAFLD/NASH. However, PPARα ligands (Clofibrate and Fenofibrate) showed no effect in inflammation and fibrosis in NASH^231^. PPARβ/δ agonist (GW501516) reduced inflammatory cells migration, insulin resistance and lipid levels, and increased ALT concentration in NASH experimental model^321^, but GW501516 has been terminated due to safety concerns. PPARγ agonists alleviated steatosis and inflammation yet with little effect fibrosis, and long time of administration is a major concern^231^. The effect of dual or pan agonists of PPARs in NASH are summarized below.

#### Elafibranor

The targets of elafibranor (GFT505) are PPARα and PPARδ^322^. Our previous results showed that GFT505 could inhibit steatosis, inflammation, and fibrosis in a NASH mouse model, and reduce the expression of lipid metabolism-, inflammation-, and fibrosis-related signaling molecules^323^. Treatment with 120 mg/d elafibranor for 1 year reduced NASH progression and liver fibrosis stage^324^. However, a phase III study of elafibranor in NASH patients was terminated because it did not achieve the predicted efficacy without safety issues (NCT02704403).

#### Saroglitazar

Saroglitazar is a novel dual PPARα/γ agonist that regulates glucose metabolism and improve insulin resistance. NAFLD/NASH patients were given placebo or 1 mg, 2 mg, or 4 mg saroglitazar. After the week 16, the ALT changes in the group taking 1 mg, 2 mg and 4 mg saroglitazine were -25.5%, 27.7%, and -45.8%, respectively, while the ALT changes in the group taking placebo were 3.4%. Administration of saroglitazar 4 mg decreased adiponectin, insulin resistance, and triglycerides, and the avarage body weight in patients taking 4 mg saroglitazar increased by 1.5 kg compared with 0.3 kg in placebo group^325^.

#### Lanifibranor

Lanifibranor (IVA337) is a PPAR α/γ/δ triple activator that can reduce immune cells infiltration and decreased steatosis in NASH experimental models^326^. In a phase 2b study, NASH patients without cirrhosis received placebo or 800 mg or 1200 mg lanifibranor daily for 24 weeks (NCT03008070). Results showed that most biomarkers of lipid, inflammation, and fibrosis were improved in both dose groups of lanifibranor. However, compared with patients receiving 800 mg lanifibranor, those receiving 1200-mg dose of lanifibranor had greater decrease in the SAF (the steatosis, activity, fibrosis) score^327^.

#### Pemafibrate

Pemafibrate targeting PPARα modulator regulates lipid and glucose metabolism. Preclinical studies have shown that pemafibrate could improve insulin resistance, inhibit hepatocyte ballooning degeneration, decrease the NAFLD score, and reduce myeloid cell recruitment^328,329^. Liver stiffness and ALT level were reduced in patients with high-risk NAFLD who received 0.2 mg pemafibrate twice daily for 72 weeks in a phase 2 trial (NCT03350165)^330^.

### Anti-fibrotic drugs targeting FXR

FXR has emerged as a promising therapeutic target for NAFLD/NASH due to its diverse functions that modulate bile acid metabolism, inflammation, and immune responses. FXR agonists could be divided into steroidal and nonsterodial, and pruritus is the most common side effect of these targeted drugs.

#### Obeticholic acid

Obeticholic acid, a steroidal FXR agonist, has been shown to improve NASH symptoms. In a phase 3 trial (NCT02548351), NASH patients were given placebo, or 10 mg or 25 mg of obeticholic acid daily. Improvement in fibrosis was achieved in 23% of patients in the obeticholic acid 25 mg group compared with 18% of patients in the 10-mg obeticholic acid group and 12% of patients in the placebo group. However, there was no difference of NASH resolution endpoint between the three groups (*P* = 0.13)^331^. Patients taking obeticholic acid usually stop or reduce their dosage because of severe pruritus.

#### Cilofexor

Cilofexor (GS-9674) is a potent and selective FXR nonsteroidal agonist which activates FXR in the intestine and does not experience enterohepatic circulation. Twenty-four weeks of cilofexor improved serum bile acids metabolism and decreased hepatic steatosis in patients with NASH, but there was no significant change in fibrosis (NCT02854605)^332^.

#### EDP-305

EDP-305 is an effective FXR agonist showing little cross reaction with other nuclear receptors. EDP-305 inhibited HSCs activation in vitro and reduced MCD-induced steatohepatitis and liver fibrosis^333^. Liver fat and ALT level were reduced in NASH patients receiving 2.5 mg of EDP-305 compared with placebo group^334^. Pruritus was also one of the most common adverse events of EDP-305^334^.

#### Tropifexor

Tropifexor is a non-steroidal FXR agonist and significantly reduced steatohepatitis and fibrosis in NASH preclinical model^335^. Tropifexor was well tolerated up to 3000 µg and 100 µg in the single- and multiple-ascending doses (SAD/MAD) studies, respectively^336^, and is currently in phase 2 development for NASH.

### Anti-fibrotic drug targeting TLR4

#### JKB-121

JKB-121 is a nonselective opioid TLR4 antagonist that has been proved to reduce LPS-induced liver inflammation in a MCD-induced model of NAFLD and inhibit the activation of HSCs^337^.

### Anti-fibrotic drugs targeting GIP and GLP-1

FXR mainly negatively regulates liver gluconeogenesis, lipogenesis, and steatosis, while GIP and GLP-1 regulates glucose and lipid metabolism by reducing appetite, regulating liver fat content and inflammation. The dual receptor agonist of GIP and GLP-1 has been considered as an important therapeutic target for NASH.

#### Tirzepatide

Tirzepatide (LY3298176), a dual GIP and GLP-1 receptor agonist, has been used to explore its efficacy in clinical trials for the treatment of NASH, obesity, and type 2 diabetes mellitus (T2DM)^338,339^. Treatment with 10 mg of tirzepatide reduced NASH-related biomarkers, such as serum ALT and aspartate aminotransferase (AST), in patients with T2DM (NCT03131687)^338^. A phase III trial investigating tirzepatide in NASH patients is currently in progress (NCT04166773).

#### Semaglutide

Semaglutide is a GLP-1 receptor agonist and has been approved for T2DM therapy. In a 72-week phase 2 trial, NASH patients with liver fibrosis of stage F1, F2, or F3 received placebo, or semaglutide at 0.1 mg, 0.2 mg, or 0.4 mg. The percentage of patients who achieved NASH improvement without worsening fibrosis was 40%, 36% and 59% in the 0.1 mg semaglutide group, 0.2 mg semaglutide group and 0.4 mg semaglutide group, respectively, and 17% in the placebo group. However, the changes in fibrosis was not statistically significant in the 0.4 mg semaglutide group (43% of the patients) and in the placebo group (33% of the patients, *P* = 0.48)^340^.

#### Cotadutide

Cotadutide (MEDI0382) is a dual receptor agonist of GIP and GLP-1 and has shown safety and tolerability^341^. Cotadutide reduced hepatic lipid content, inflammation, steatosis, and NAS score in a mouse model of NASH^342^.

### Anti-fibrotic drugs targeting CFTR

Drugs that improve the structure and function of CFTR have good therapeutic prospects in cystic fibrosis. At present, two kinds of drugs with different action mechanisms but complementary therapeutic effects have been developed, namely, CFTR potentiators and CFTR correctors^343^. CFTR potentiators enhance the gating of CFTR at the cell surface to mediate ion transport and are very effective in treating gated mutations^344^. CFTR correctors modify the processing and transportation of CFTR protein in cells, thus increasing the number of functional CFTR on the cell surface^345^.

#### Ivacaftor and Tezacaftor

Ivacaftor (VX-770) is the first CFTR potentiators approved by the FDA for cystic fibrosis patients with the gated mutation. Tezacaftor is a CFTR corrector approved by the FDA to be utilized in combination with ivacaftor. In a phase 2 clinical study, daily intake of 100 mg tezacaftor and 150 mg ivacaftor every 12 hours was effective in reducing chloride ion concentration in the sweat of cystic fibrosis patients, while increasing the percent predicted FEV1 (ppFEV1) value by 3.75% (NCT01531673)^346^.

#### Lumacaftor

Lumacaftor (VX-809), a CFTR corrector, is usually used in combination with ivacaftor for the treatment of cystic fibrosis. Lumacaftor increased the trafficking of CFTR protein to the extracellular membrane, while ivacaftor enabled the opening of dysfunctional chloride channels^347^. In 6- to 11-year-old patients with cystic fibrosis, sweat chloride concentration and CFQ-R RD score were improved after lumacaftor/ivacaftor combination therapy, but the FEV1 parameter was not changed (NCT02514473)^348^. However, FEV1 increased in patients with cystic fibrosis aged 12 years or older in a combination therapy with lumacaftor and ivacaft (NCT01807949)^349^.

#### ABBV-2222

ABBV-2222 (GLPG2222) is a novel and potent CFTR corrector^350^. Oral administration of ABBV-2222 once daily for 29 days in patients with homozygous or heterozygous of F508del CFTR and a gating mutation reduced sweat chloride concentrations in a dose-dependent manner without ppFEV1 improvements (NCT03119649 and NCT03045523)^351^.

#### Eluforsen

Eluforsen is an antisense oligonucleotide targeting the F508del mutation mRNA region to restore CFTR function^352^. Inhalation of eluforsen by single or multiple doses (up to 50 mg) demonstrated safety and tolerability^353^. In a phase 1b study, cystic fibrosis patients with a FEV1 > 70% in four single ascending dose cohorts and four MAD cohorts received eluforsen three times weekly for 4 weeks. CFQ-R Respiratory Symptom Score was improved in subjects of three groups in the MAD study^353^.

## Conclusions

The high mortality and complex pathogenesis of fibrotic diseases pose great challenges in clinical therapy. Various cells and signaling pathways are involved in the progression of fibrosis. Drugs targeting these abnormal pathways are constantly being developed, and most of them demonstrate good anti-fibrotic properties in clinical trials. However, the side effects of these drugs often lead to drug discontinuation. Therefore, reducing adverse effects is also a great challenge for drug development. In addition, due to the complicated interaction of these signaling pathways in fibrosis, multitarget drug regimens would be beneficial for fibrosis therapy. In conclusion, this review provides reference for further mechanism and drug study of fibrosis.
